# Clinical performance of AMA‐M2, anti‐gp210 and anti‐sp100 antibody levels in primary biliary cholangitis: When detected by multiplex bead‐based flow fluorescent immunoassay

**DOI:** 10.1002/iid3.1161

**Published:** 2024-01-19

**Authors:** Zhan Wang, Yongxin Li, Lisheng Ren, Yujie Li, Tiantian Xu, Wenshuai Li, Weize Gao, Guirong Sun, Mingjun Liu

**Affiliations:** ^1^ Department of Clinical Laboratory, Key Laboratory of Laboratory Medicine The Affiliated Hospital of Qingdao University Qingdao China; ^2^ Qingdao Women and Children's Hospital Qingdao University Qingdao China

**Keywords:** autoantibodies level, diagnosis, multiplex bead‐based flow fluorescent immunoassay array, primary biliary cholangitis

## Abstract

**Background and Aim:**

Primary biliary cholangitis (PBC) is a chronic autoimmune cholangiopathy, characterized by the presence of some autoantibodies in the serum. This study aimed to evaluate the clinical significance of AMA‐M2, anti‐gp210 and anti‐sp100 antibody levels detected by multiplex bead‐based flow fluorescent immunoassay (MBFFI) in PBC.

**Methods:**

This study cohort included 238 PBC patients, 81 autoimmune hepatitis (AIH) patients, 62 systemic lupus erythematosus (SLE) patients, and 118 healthy controls. Serum AMA‐M2, anti‐gp210 and anti‐sp100 antibody were detected by MBFFI and immunoblotting assay (IBT). The relationship between three antibody levels and cirrhosis, liver function, cholestasis markers and therapeutic effect to ursodesoxycholic acid (UDCA) was evaluated in PBC.

**Results:**

MBFFI were presented good coincidence rate (87.39%–95.38%) with IBT. The level of AMA‐M2, anti‐gp210 and anti‐sp100 antibodies in PBC patients were higher than other disease group and healthy controls (*p* ＜ .01). When compared with the healthy controls group, the AUC of AMA‐M2, anti‐gp210 and anti‐sp100 antibodies were 0.9245, 0.7619, and 0.6789, respectively. In addition, gp210 antibody levels have diagnostic value in patients with liver cirrhosis (AUC: 0.7567). We found that when combine detect these three antibodies, the sensitivity was higher than individually detection. High level of serum anti‐gp210 antibody could be related to worse liver function and more severe cholestasis in PBC patients. Moreover, serum antibody levels may decrease or remained flat in patients who responded well to UDCA.

**Conclusion:**

The detection of AMA‐M2, anti‐gp210 and anti‐sp100 antibody levels by MBFFI showed good performance in the diagnosis of PBC. Serum anti‐gp210 antibody level is related to cirrhosis, poor liver function and severe cholestasis in PBC.

## Graphical abstract

The aim of this study was to evaluate the clinical significance of quantitative detection of AMA‐M2, anti‐gp210 and anti‐SP100 antibody levels in PBC.

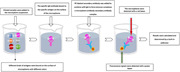

## 1 INTRODUCTION

Primary biliary cholangitis (PBC) is a chronic, cholestatic autoimmune liver disease characterized by progressive inflammatory destruction of small intrahepatic bile ducts.^1^, ^2^ Serum antibodies detection is an important step in the diagnosis of PBC.^3^, ^4^ Anti‐mitochondrial antibody (AMA) is an important serological hallmark for the diagnosis of PBC and is typically observed in more than 90% of patients.^5^, ^6^ Besides, antinuclear antibodies (ANA) are present in part of PBC patients, especially nuclear envelope antibody (anti‐gp210 antibody) and multiple nuclear dots (anti‐sp100 antibody).^7^, ^8^ Some studies have shown that nearly 30% to 50% of PBC patients are positive for anti‐gp210 and anti‐sp100 antibodies.^9^, ^10^

At present, immunoblotting assay (IBT) is widely used method to detect autoantibodies in China. IBT has some disadvantages of being cumbersome and time‐consuming. Multiplex bead‐based flow fluorescent immunoassay (MBFFI) is a latest technology developed to detect autoantibodies. Compared with the traditional qualitative detection, it has the advantages of high throughput, high sensitivity, high automation and good stability. In other autoimmune diseases, the autoantibody level is of great value in the evaluation of disease progression, monitoring of curative effect and prognosis.^11^, ^12^, ^13^ However, the clinical significances of AMA‐M2, anti‐gp210 and anti‐sp100 antibody levels are not clear in PBC.

In this study, we compared the differences between MBFFI and IBT to evaluate the detection performance of MBFFI. The clinical significance of AMA‐M2, anti‐gp210 and anti‐sp100 antibody levels was evaluated in PBC.

## 2 MATERIALS AND METHODS

### 2.1 Study population

This study included 238 PBC patients which were visited The Affiliated Hospital of Qingdao University from August 2018 to August 2022. The diagnostic criteria of PBC are as follows: (i) The serum level of alkaline phosphatase (ALP) were elevated and the titer of AMA was ≥1:40 in adult patients with cholestasis; (ii) Specific ANA (nuclear dots or perinuclear rims) can be detected in the serum of AMA negative patients; (iii) Liver biopsy is not recommended for diagnosis unless with the lack of PBC‐specific antibodies or coexistent autoimmune liver disease (AIH), nonalcoholic fatty liver disease (NASH) and other systemic diseases.^3^ All patients fulfilled at least two of these three criteria. The patient concomitant of virus hepatitis, human immunodeficiency virus infection, alcoholic intemperance, drug hepatitis, biliary obstruction, AIH‐PBC overlap syndrome, tumor, or liver transplant were excluded. According to the results of B‐ultrasound, CT and other imaging examinations, PBC patients were further divided into cirrhosis group and non‐cirrhosis group.

Other disease groups included 81 patients with autoimmune hepatitis (AIH) and 62 patients with systemic lupus erythematosus (SLE) who visited The Affiliated Hospital of Qingdao University at the same time, and the inclusion criteria were all based on the diagnostic guidelines.^14^, ^15^ There were 118 health subjects in the healthy control group. The samples of the healthy control group were from the healthy individuals who underwent physical examinations at the Affiliated Hospital of Qingdao University. The age and gender of all healthy subjects were matched with PBC patients, and the indicators of liver function were within the reference range. There was no obvious abnormality in liver imaging examination. People with organic diseases or without organic diseases but receiving drug treatment were excluded. The research related to human use has been complied with all the relevant national regulations, institutional policies and in accordance the tenets of the Helsinki Declaration, and has been approved by the hospital ethics committee. Written informed consent was obtained from all the participants before the enrollment of this study.

### 2.2 The sample collection

The remaining serum of the subjects after completion of routine testing was collected. A total of 200 μL of serum was retained for each subject, frozen in a −36°C refrigerator, and tested after thawing. All samples were tested within 1 week after collection.

### 2.3 Qualitative detection of antibodies

AMA‐M2, anti‐gp210 and anti‐sp100 antibodies were detected qualitatively by IBT. The IBT assay used EUROBlotMaster II (EUROIMMUN) and EUROLINE Autoimmune Liver Disease (IgG) kit. The antigens coated by the membrane strip mainly included AMA‐M2, anti‐gp210, anti‐sp100, SLA, LC‐1, LKM‐1. The membrane strips were placed in a warm bath, and the sample serum was diluted 1:100 and added to the warm bathe; Shaked the warm bath for 30 min to fully combine them, and then washed after full reaction; The enzyme conjugates were added in warm bath, after 30 min, washed the membrane strips with diluted washing buffer for three times, 5 min/time; Finally, 1.5 mL of substrate solution was added to a warm bath for color development. After drying, we used the EUROBLineScan software to interpret the results. All operations must follow the standard operating procedures.

### 2.4 Quantitative detection of antibodies

**Figure 1:**
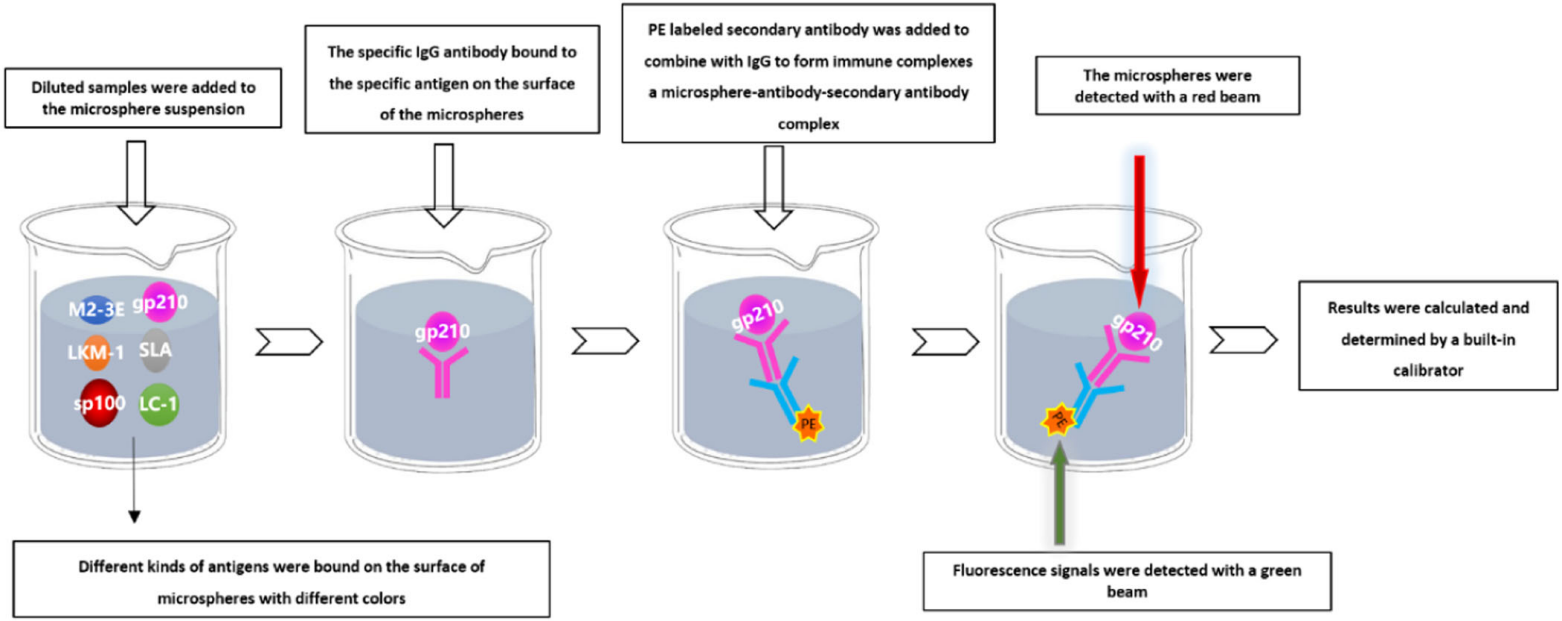
The principle and major steps of MBFFI assay. PE, phycoerythrin.

Quantitative detection of antibodies was performed using multiplex bead‐based flow fluorescent immunoassay (MBFFI). Among them, TESMI F3999 Automatic sampler Luminex 200TM and detection reagents are provided by Tellgen Co., Ltd. The autoimmune liver disease antibody kit was used to detect antibodies in all serum samples, and the whole process consisted of a two‐step incubation process. The detailed operation protocol was shown in Figure 1. Antibody levels greater than 20 AU were defined as antibody positive.

### 2.5 Detection methods of other laboratory indicators

Serum alanine aminotransferase (ALT), aspartate aminotransferase (AST), ALP, gamma‐glutaryltransferase (GGT), total bilirubin (TBIL), direct bilirubin (DBIL) and albumin (ALB) in PBC patients was detected by Hitachi 7600 series automatic biochemical analyzer (Japan).

### 2.6 Statistical analyses

Kolmogorov‐Smirnov test was used to the normality of continuous variable data. Non‐normally distributed data were presented as median and interquartile range, and comparisons were made between groups using the Mann‐Whitney U‐test. The Kruskal‐Wallis test was used to compare three or more quantitative data groups and then compared them pairwise. The receiver operating characteristics (ROC) curve and area under the curve (AUC) were calculated to identify differences between the groups. Spearmen correlation analysis was used for continuous data. Kappa test was used to assess consistency. Statistical analyses were performed with The Social Sciences version 22.0 software (SPSS Inc.) and The Graph Pad Prism Version 8.0.1 (244) software (Inc.). *p* < .05 was considered statistically significant.

## 3 RESULTS

### 3.1 General characteristics of the subjects

PBC patients include 224 females and 14 males, with mean age of 57.01 ± 11.16 years old and range 24–91. The 24 patients were newly diagnosed, and the remaining 214 patients were treated with ursodesoxycholic acid (UDCA). A total of 143 other disease serum samples were analyzed, of which 81 were from patients with AIH and 62 were from patients with SLE. AIH patients include 69 females and 12 males (mean age 53.35 ± 13.47, range 11–81); SLE patients include 54 females and 9 males (mean age 52.71 ± 12.91, range 24–79). There were 118 health subjects in the healthy control group, including 99 females (83.89%) with a median age of 53 years and a range of 32–75 years.

### 3.2 Comparison of the detection consistency between IBT and MBFFI

In IBT, the positive rates of AMA‐M2, anti‐gp210 and anti‐sp100 antibodies were 81.93%, 35.71%, and 21.85%, respectively. According to MBFFI detection results, the positive rates of AMA‐M2, anti‐gp210 and anti‐sp100 antibodies were 85.72%, 34.03%, and 26.47%, respectively (Table 1). The coincidence rate of two methods for detecting antibodies were assessed in PBC patients. As observed in Table 2, MBFFI detection results were presented good coincidence rate (87.39%‐95.38%) with IBT detection. The two methods had strong consistency for the detection of anti‐sp100 antibodies (kappa = 0.821, *p* ＜ .001), and had moderate consistency of AMA‐M2 and gp210 antibodies (kappa = 0.894 and 0.729, respectively; all *p* ＜ 0.001).

**Table 1:** Consistency analysis of three antibodies tested by IBT and MBFFI.

Antibody	Positive rate, *n* (%)		Negative rate, *n* (%)		Consistency	Kappa	*p* value
	IBT	MBFFI	IBT	MBFFI			
AMA‐M2	195 (81.93%)	204 (85.72%)	43 (18.07%)	34 (14.29%)	92.02%	0.706	.000
anti‐gp210	85 (35.71%)	81 (34.03%)	153 (64.29%)	157 (65.97%)	87.39%	0.723	.000
Anti‐sp100	52 (21.85%)	63 (26.47%)	186 (78.15%)	175 (73.53%)	95.38%	0.874	.000

**Table 2:** AMA‐M2, anti‐gp210 and anti‐sp100 levels in PBC patients, other disease patients and healthy controls.

Antibody category	PBC (*n* = 238)		Other disease (*n* = 143)	Healthy controls (*n* = 118)
	cirrhosis (*n* = 53)	noncirrhosis (*n* = 185)		
AMA‐M2 AU	188.33 (91.44–245.03)^a^^,^ c	192.46 (50.46–265.25)^a^^,^ c	4.03 (1.77–10.48)	3.48 (1.52–6.36)
anti‐gp210 AU	67.91 (5.83–172.74)^a^^,^ b ^,^ c	3.57 (2.24–19.13)^a^^,^ c	2.45 (1.65–4.61)	2.08 (1.34–3.84)
anti‐sp100 AU	2.13 (1.52–10.60)^a^^,^ c	2.51 (1.77–10.48)^a^	1.82 (1.30–6.36)	1.60 (1.11–2.80)

^a^ *p* < .01 versus other disease group.

^b^ *p* < .01 versus noncirrhosis group.

^c^ *p* < .01 versus healthy controls.

### 3.3 Antibody patterns of patients with PBC

**Figure 2:**
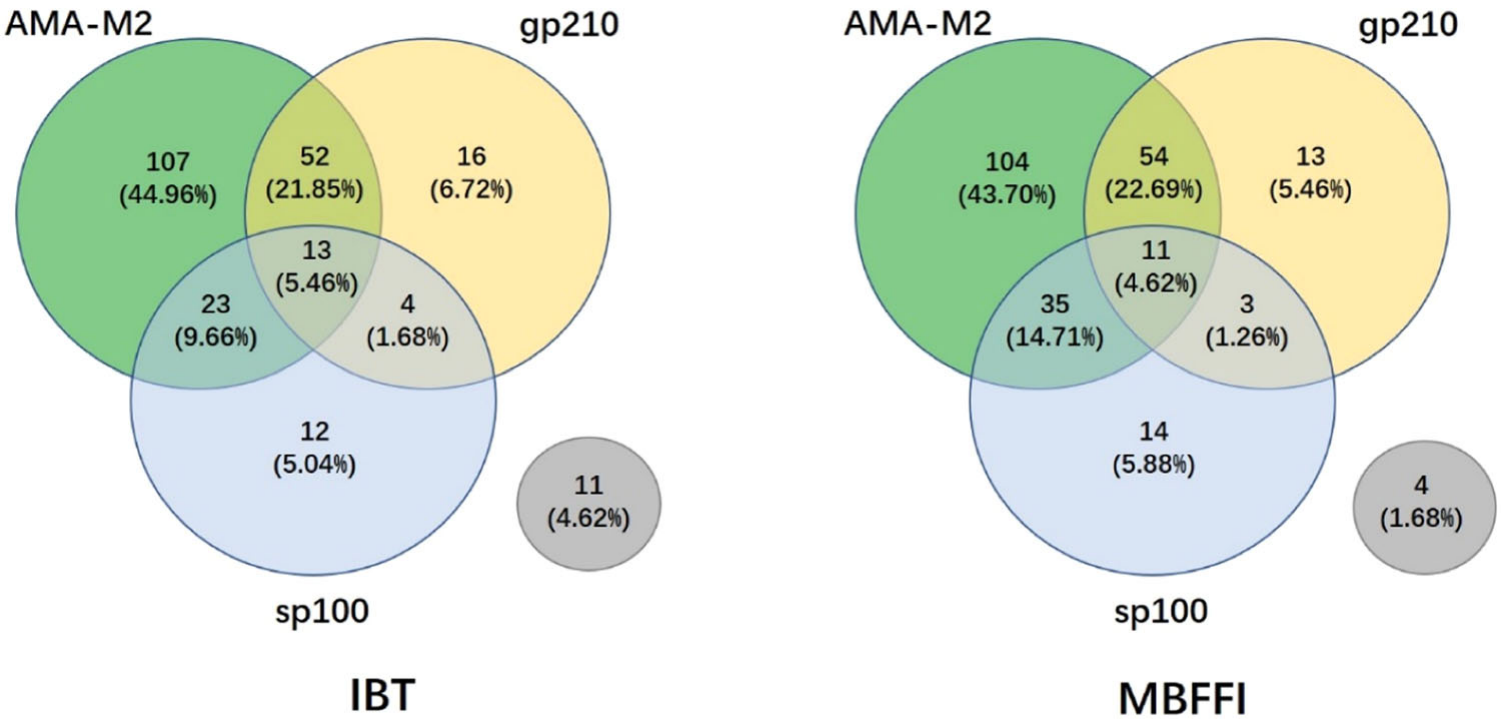
Venn diagram shows the antibody pattern of PBC patients detected by IBT and MBFFI.

The pattern of three antibodies in patients with PBC was presented in the Venn diagram (Figure 2). Among the two methods (MBFFI vs. IBT), the most frequently observed antibody pattern was AMA‐M2 single positive (43.70% vs. 44.96%). The secondary antibody pattern was AMA‐M2 and anti‐gp210 antibodies double positive (22.69% vs. 21.85%). The proportion of patients who were simultaneously positive for AMA, anti‐gp210 and anti‐sp100 antibodies were 4.62% and 5.46%, respectively. In MBFFI detection, the proportion of PBC patients who were negative for all antibodies was 1.68%, which was lower than IBT detection (4.62%).

### 3.4 Serum levels and diagnostic performance of AMA‐M2, anti‐gp210 and anti‐sp100 antibodies in PBC patients

As expected, the level of AMA‐M2, anti‐gp210 and anti‐sp100 antibodies in PBC patients were higher than AIH patients, SLE patients (Other disease group, OD) and healthy controls (HC) (*p* ＜ .01). There was no significant difference in the level of three antibodies between OD group and HC group. The level of serum gp210 antibody in cirrhosis group was higher than that in non‐cirrhosis group (*p* ＜ .01). There was no significant difference in AMA‐M2 and sp100 antibody levels between cirrhosis and non‐cirrhosis groups (Table 2).

**Figure 3:**
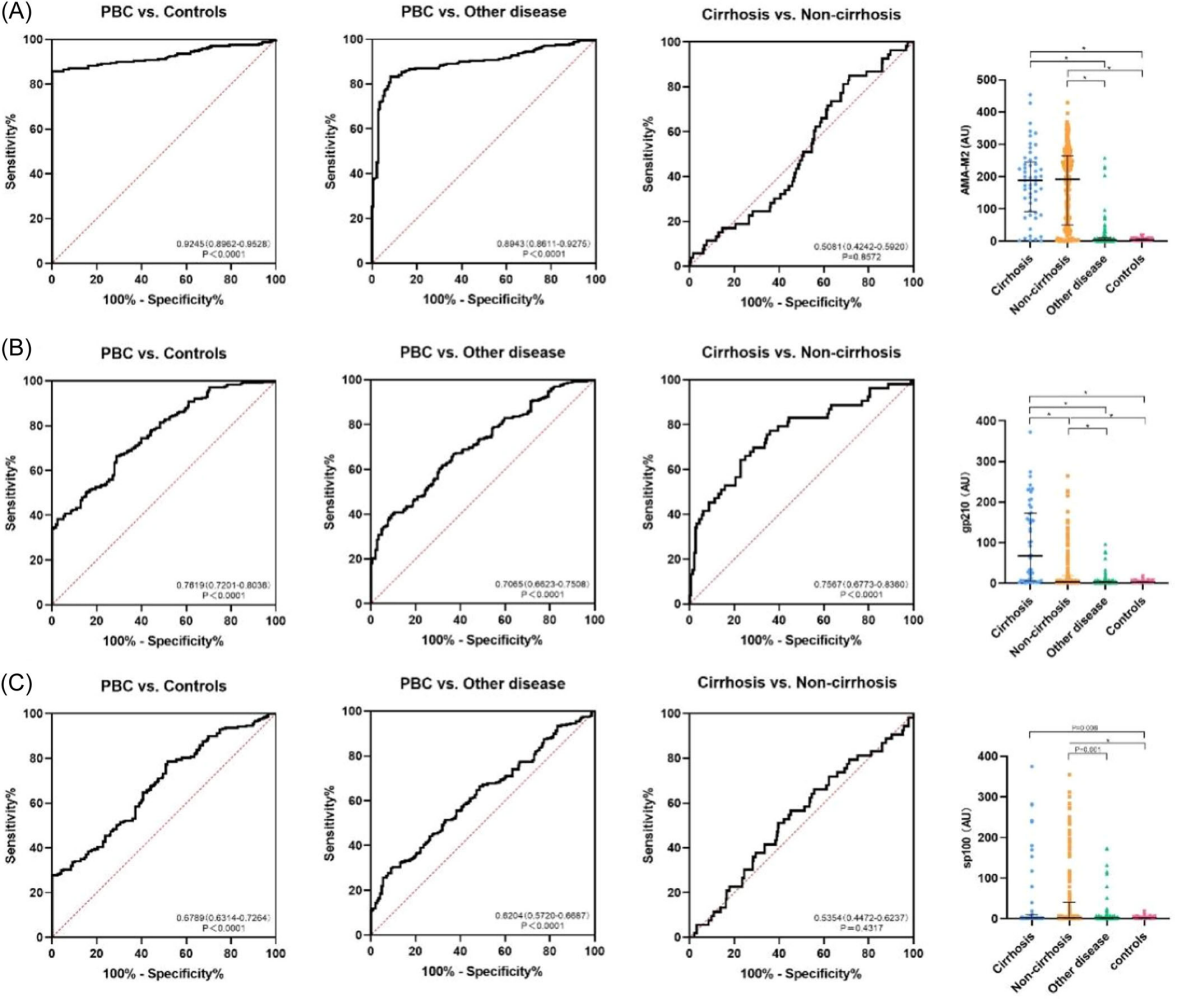
Diagnostic performances of AMA‐M2, anti‐gp210 and anti‐sp100 antibodies in PBC and PBC cirrhosis.

We evaluated the diagnostic performance of AMA‐M2, anti‐gp210 and anti‐sp100 antibodies by using healthy people and other disease groups as controls, as shown in Figure 3. When compared with the healthy controls group, the AUC of AMA‐M2, anti‐gp210 and anti‐sp100 antibodies were 0.9245, 0.7619 and 0.6789, respectively. The area under the curve of AMA‐M2, anti‐gp210 and anti‐sp100 antibodies were 0.8943, 0.7065 and 0.6204 when the control was the other disease group.

In addition, we also evaluated the ability of anti‐gp210 to diagnose cirrhosis. The area under the curve of gp210 was (0.7567), which was higher than that of AMA‐M2 (0.5081) and sp100 (0.5354).

### 3.5 Diagnostic value of three antibodies individual or combined detection by MBFFI in PBC patients

Taking healthy subjects as controls, we evaluated the diagnostic performance of the MBFFI method for PBC (Table 3). The sensitivity of AMA‐M2 (85.71%) was higher than that of anti‐gp210 (34.03%) and anti‐sp100 (26.47%) antibodies, but the specificity was relatively low. The Youden index of AMA‐M2 was 0.78, which was higher than that of anti‐gp210 (0.31) and anti‐sp100 (0.22) antibodies. We attempted to enhance the sensitivity of the assay using several panels of antibodies, and combinational detection showed the highest sensitivity in the three antibodies (98.32%). Anti‐gp210 antibody had the best PPV for PBC, reaching 91.01%.

**Table 3:** Diagnostic value of three antibodies individual or combined detection by MBFFI in PBC patients.

Panel of antibody	Positive, *n* (%)		Se (%)	Sp (%)	YI	PPV (%)	NPV (%)	LR+	LR−
	PBC	Controls							
AMA‐M2	204 (85.71%)	21 (7.98%)	85.71%	92.02%	0.78	90.67%	87.68%	10.73	0.16
gp210	81 (34.03%)	8 (3.04%)	34.03%	96.96%	0.31	91.01%	61.89%	11.19	0.68
sp100	63 (26.47%)	11 (4.18%)	26.47%	95.82%	0.22	85.14%	59.02%	6.33	0.77
AMA‐M2+gp210	220 (92.44%)	26 (9.89%)	92.44%	90.11%	0.83	89.43%	92.94%	9.35	0.08
AMA‐M2+sp100	221 (92.86%)	26 (9.89%)	92.86%	90.11%	0.83	89.47%	93.31%	9.39	0.08
gp210+sp100	130 (54.62%)	17 (6.46%)	54.62%	93.54%	0.48	88.44%	69.49%	8.45	0.49
AMA‐M2+gp210+sp100	234 (98.32%)	31 (11.79%)	98.32%	88.21%	0.87	88.30%	98.31%	8.34	0.02

Abbreviations: LR− , negative likelihood ratio; LR+ , positive likelihood ratio; NPV, negative predictive value; PPV, positive predictive value; Se, sensitivity; Sp, specificity; YI, Youden Index.

### 3.6 The correlation between serum antibody levels and laboratory indices in PBC

The correlation between serum level of AMA‐M2, anti‐gp210, and anti‐sp100 antibodies and laboratory indices were assessed in patients of PBC (Table 4). Serum AMA‐M2 level was positively correlated with ALT (*r* = .254, *p* = .022) and ALP (*r* = .306, *p* = .009), but no significant correlation was found with other biochemical indicators. Serum anti‐gp210 antibody level was positively correlated with ALT (*r* = .228, *p* = .041), AST (*r* = .356, *p* = .001), TBIL (*r* = .320, *p* = .015), DBIL (*r* = .359, *p* = .010), ALP (*r* = .305, *p* = .010) and GGT (*r* = .288, *p* = .014), while negatively correlated with ALB (*r* = −0.350, *p* = .007). Similarly, there was no correlation between serum sp100 antibody level and laboratory indices.

**Table 4:** Correlation between AMA‐M2, gp210, sp100 antibody levels and laboratory indices in PBC patients.

laboratory indices	*n*	AMA‐M2		gp210		sp100	
		*r*	*p*	*r*	*p*	*r*	*p*
ALT		.254	.022*	.228	.041*	.041	.700
AST		.198	.077	.356	.001*	.072	.505
TBIL		.089	.512	.320	.015*	−.116	.373
DBIL		.015	.916	.359	.010*	−.059	.674
ALP		.306	.009*	.305	.010*	−.184	.108
GGT		.231	.050	.288	.014*	−.072	.530
ALB		.053	.693	−.350	.007*	.094	.459

Abbreviations: ALT, alanine aminotransferase; AST, aspartate aminotransferase; ALP, alkaline phosphatase; ALB, albumin; DBIL, direct bilirubin; GGT, gamma‐glutaryl transferase; TBIL, total bilirubin.

* *p* ＜ .05.

### 3.7 AMA‐M2, anti‐gp210 and anti‐sp100 antibody levels in PBC patients after treatment

**Figure 4:**
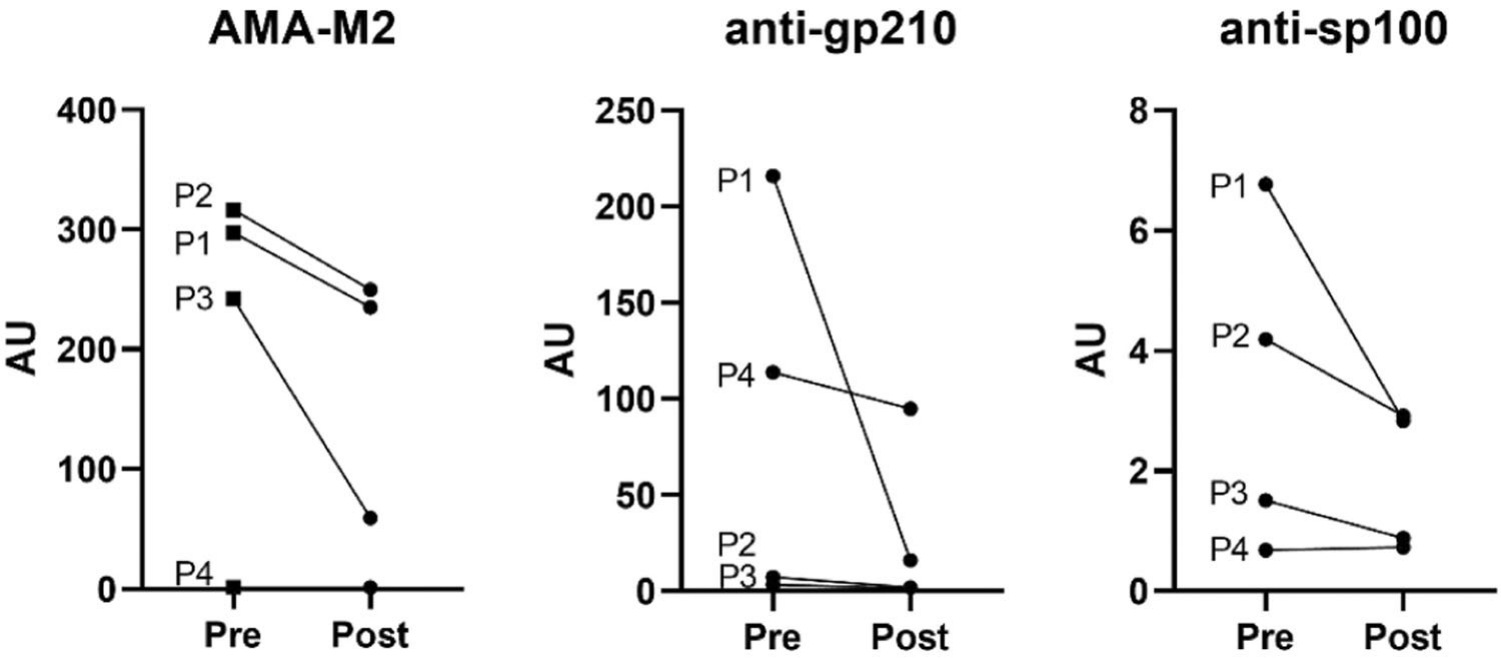
AMA‐M2, anti‐gp210 and anti‐sp100 antibodies measured by quantitative MBFFI in newly‐diagnosed PBC patients at pre and posttreatment. P, patient; Pre, before UDCA treatment; Post, after UDCA treatment.

Four patients with newly diagnosed PBC were followed up for at least 6 months. All the 4 patients were treated with the same amount of UDCA and no other drugs such as hormones were used. MBFFI method was used to detect serum antibody levels before and after treatment. The antibody levels of these 4 patients before and after treatment are shown in the Table 5. In these patients, decreased levels of three autoantibodies were observed after the treatment (Figure 4).

**Table 5:** Antibody levels of four4 newly diagnosed PBC patients before and after treatment.

Patient No.	Follow up time, mouth	AMA‐M2 AU		Anti‐gp210 AU		Anti‐sp100 AU	
		Pre	Post	Pre	Post	Pre	Post
1	12	297.52	235.29	215.91	16.22	6.77	2.84
2	6	316.57	249.96	7.31	2.08	4.19	2.92
3	15	242.21	59.06	3.52	1.50	1.51	0.88
4	6	1.03	1.01	113.79	94.94	0.68	0.73

Abbreviations: Pre, before UDCA treatment; Post, after UDCA treatment.

## 4 DISCUSSION

The detection of autoantibodies is very important for the diagnosis and prognosis of PBC. According to several most recent studies, autoantibody levels may change during the course of the disease,^16^, ^17^, ^18^ but qualitative tests may not be sensitive to such changes. In this study, excluding a small number of inconsistencies due to methodological reasons or subjective reasons, the overall consistency of the two methods was high, suggesting that MBFFI can be used to identify patients with PBC. Quantitative detection can quickly assess antibody level changes in real time, which can directly reflect the disease state and help clinicians personalized treatment. Therefore, MBFFI may become a commonly used clinical method for the detection of liver disease‐related autoantibodies. These results would be a fundamental guidance for clinicians to establish treatment strategies in the transition of liver disease‐related autoantibodies detection methods.

In this study, we observed that serum AMA‐M2 level in PBC patients were significantly higher than those in other disease groups and healthy controls. The E2 subunit of pyruvatedehydrogenase complex (PDC‐E2) is one of the antigen targets of AMA‐M2 and belongs to the mitochondrial enzymes located in the innermitochondrial membrane.^19^ Some studies have shown that when the bile duct epithelial cells (BECs) initiate their own apoptosis, the antigen epitopes of PDC‐E2 can be exposed.^20^ Under the stimulation of PDC‐E2, the immune cells will be activated and attack adjacent BECs, resulting in a series of immune cascade reactions against BEC.^21^, ^22^, ^23^ Therefore, high level of AMA‐M2 can be detected in serum of PBC patients, this result is also consistent with the study of Han et al.^24^ Serum level of anti‐gp210 and anti‐sp100 antibodies in PBC group were significantly higher than those in other disease groups and healthy controls, while there was no significant difference between other disease and healthy control groups. Hu et al. showed that in the Chinese population, the detection rates of anti‐gp210 and anti‐sp100 antibody in serum of AIH patients were 7% and 16%, respectively.^25^ In this study, although the serum level of anti‐gp210 and anti‐sp100 antibodies in some patients with other diseases were higher than cut‐off values, they were much lower than those in the PBC group. Besides, three antibody levels also performed well diagnostic value in PBC, which is consistent with previous study.^26^ Previous studies have shown that the seropositive of anti‐gp210 antibody may indicate the progressive disappearance of the intrahepatic bile duct in a short period of time and the aggravation of hepatic fibrosis.^27^, ^28^ In this research, gp210 antibody level was higher in PBC patients with cirrhosis than those with non‐cirrhosis. Accordingly, PBC patients with high‐level of gp210 antibody is more likely to occur cirrhosis than patients with low‐level of gp210 antibody and it requires close monitoring serum level of gp210 antibody by the clinicians during treatment.

We further analyzed the diagnostic performance of MBFFI in detecting AMA‐M2, anti‐gp210 and anti‐sp100 antibodies. Villalta et al.^29^ applied a microsphere‐based multiplex analysis system of INOVA Diagnostics to detect three autoantibodies. The AUC of AMA‐M2, anti‐gp210 and anti‐sp100 antibodies detected by that method were 0.836, 0.528, and 0.487 respectively. The AUC of AMA‐M2, anti‐gp210 and anti‐sp100 antibodies in our study were 0.8943, 0.7065, and 0.6204 when the other disease group was the other disease group. There may be two reasons for this discrepancy: first, there were geographic and ethnic differences in the subjects of the two studies; second, the control group included different types of patients. In addition, we found that the AUC and sensitivity of the combined detection of antibodies in the diagnosis of PBC were significantly improved compared with those of the single detection, suggesting that we can improve the diagnostic rate of PBC through the combined detection of antibodies.

To date, the role of AMA in evaluating disease progression is still controversial. Gatselis et al. believed that the fluctuation of AMA was correlated with disease severity.^18^ On the contrary, Norstrand et al. believed that AMA was not useful parameters for predicting disease progression in patients with PBC.^30^ In our study, the level of AMA‐M2 antibody was positively correlated with the levels of serum ALT, ALP and GGT, which reflect hepatocyte damage and cholestasis. These results suggest that the degree of cholestasis and hepatocyte injury in PBC patients would worsen with the increase of AMA level. Flisiak et al. found that AMA level were correlated with bilirubin and ALB.^31^ However, this correlation was weak in our study. The reason for this difference could be related to the small number of PBC patients with cirrhosis in this study. In addition, with the increase of serum anti‐gp210 antibody level, the levels of serum ALT, AST, TBIL, DBIL, ALP and GGT also increased significantly, while the level of ALB decreased. Our data are consistent with the results of previous semi‐quantitative assays.^18^, ^32^ Evidence showed that the presence of anti‐gp210 antibody was associated with adverse phenotypes of PBC and it may be a predictor of poor prognosis.^33^, ^34^ Our findings have indicated that high level of serum gp210 antibody could be related to worse liver function and more severe cholestasis in PBC patients.

Whether serum antibody levels in PBC patients will change with treatment is currently inconclusive. According to Joshi et al.^35^ research, serum AMA antibody titers in PBC patients do not change over time, so it cannot be used to predict disease progression. The Heseltine^36^ study believes that there is a correlation between the level of AMA and biochemical prognostic indicators such as bilirubin, and it has been confirmed that it has a certain relationship with histological progress. In this study, 4 newly diagnosed patients were followed up, and we found that AMA‐M2 levels showed a downward trend during treatment. These four patients responded well to the UDCA and did not experience rapid disease progression in the short term. Anti‐gp210 remained an independent factor associated with worse outcome in PBC. Nakamura et al. believed that the persistence of anti‐gp210 after UDCA treatment is a risk factor for the progression to end‐stage hepatic failure, whereas the disappearance of anti‐gp210 after therapy indicate a more favorable clinical course in PBC.^37^ The above results are consistent with our findings. The role of anti‐sp100 antibody levels in disease detection and treatment is still controversial. Gatselis et al.^18^ conducted a 3‐year follow‐up of 14 anti‐sp100 antibody‐positive PBC patients and showed that reduced anti‐sp100 titers were associated with improved Mayo risk score and UDCA response. Tana et al.^2^ study showed that decreased levels of anti‐sp100 antibodies were associated with increased fibrosis. It is suggested that the change of anti‐sp100 antibody level has a certain predictive effect on the progress of the disease. Our data could indicate that serum antibody levels were decreased or remained flat in patients who responded well to UDCA.

A limitation of this study is lack of newly diagnosed patients in the study cohort，and fewer patients are followed up. We will continue to follow up newly diagnosed patients for a long time to evaluate the relationship between autoantibody levels and the effect of UDCA treatment. In addition, the relationship between baseline antibody levels and disease prognosis needs to be evaluated.

In conclusion, the detection of AMA‐M2, anti‐gp210 and anti‐sp100 antibody levels by MBFFI showed good performance in the diagnosis of PBC. Serum anti‐gp210 antibody level is related to cirrhosis, poor liver function and severe cholestasis in PBC.

## AUTHOR CONTRIBUTIONS

**Zhan Wang**: Conceptualization; formal analysis; investigation; methodology; visualization; writing—original draft. **Yongxin Li**: Conceptualization; data curation; formal analysis; investigation; methodology; visualization; writing—original draft. **Lisheng Ren**: Methodology. **Yujie Li**: Formal analysis; investigation. **Tiantian Xu**: Formal analysis; investigation. **Wenshuai Li**: Formal analysis; investigation. **Weize Gao**: Formal analysis; investigation. **Guirong Sun**: Investigation; supervision; writing—review and editing. **Mingjun Liu**: Conceptualization; funding acquisition; methodology; project administration; resources; writing—review and editing.

## CONFLICT OF INTEREST STATEMENT

The authors declare that they have no competing interests regarding the publication of this paper.

## ETHICS STATEMENT

Research involving human subjects complied with all relevant national regulations, institutional policies and is in accordance with the tenets of the Helsinki Declaration (as revised in 2013), and has been approved by the authors' Institutional Review Board (QYFYWZLL27320). Written informed consent was obtained from all the participants before the enrollment of this study.

## Supporting information

Supporting information.

## Data Availability

The data that support the findings of this study are available upon reasonable request from the corresponding author. The data are not publicly available due to privacy and ethical restrictions.
